# From Lévy to Brownian: A Computational Model Based on Biological Fluctuation

**DOI:** 10.1371/journal.pone.0016168

**Published:** 2011-02-03

**Authors:** Surya G. Nurzaman, Yoshio Matsumoto, Yutaka Nakamura, Kazumichi Shirai, Satoshi Koizumi, Hiroshi Ishiguro

**Affiliations:** 1 Graduate School of Engineering, Osaka University, Suita, Japan; 2 National Institute of Advanced Science and Technology (AIST), Tsukuba, Japan; 3 Graduate School of Engineering Science, Osaka University, Toyonaka, Japan; Dana-Farber Cancer Institute, United States of America

## Abstract

**Background:**

Theoretical studies predict that Lévy walks maximizes the chance of encountering randomly distributed targets with a low density, but Brownian walks is favorable inside a patch of targets with high density. Recently, experimental data reports that some animals indeed show a Lévy and Brownian walk movement patterns when forage for foods in areas with low and high density. This paper presents a simple, Gaussian-noise utilizing computational model that can realize such behavior.

**Methodology/Principal Findings:**

We extend Lévy walks model of one of the simplest creature, *Escherichia coli*, based on biological fluctuation framework. We build a simulation of a simple, generic animal to observe whether Lévy or Brownian walks will be performed properly depends on the target density, and investigate the emergent behavior in a commonly faced patchy environment where the density alternates.

**Conclusions/Significance:**

Based on the model, animal behavior of choosing Lévy or Brownian walk movement patterns based on the target density is able to be generated, without changing the essence of the stochastic property in *Escherichia coli* physiological mechanism as explained by related researches. The emergent behavior and its benefits in a patchy environment are also discussed. The model provides a framework for further investigation on the role of internal noise in realizing adaptive and efficient foraging behavior.

## Introduction

It has been noticed that in nature many predators do random search as they have to make foraging, searching for foods, decision with little, if any, knowledge of present resource distribution and availability [1]. This feature leads to a question of: “what is the most efficient statistical strategy to optimize a random search?” It is shown that for sparse targets (i.e low target density), the efficiency, defined as number of targets (e.g. preys, foods) found divided by the traveled distance, is maximized when the flight lengths follows an inverse power law distribution with a heavy tail: a Lévy walk [2]. Although it has been revised for certain animals [3], experimental data shows that the movement patterns of various animals like spider monkeys, goats, solitary fallow deer, fruit files, zooplanktons and various marine predators like sharks, sea turtles and penguins fits a Lévy walk statistic [4]–[10].

Further theoretical study shows that while Lévy walks is more efficient for a search with sparse, smaller and slower targets, the opposite conditions shows Brownian walks as the favorable strategy [11]. In a patchy environment where the target density alternates, other results also show that performing Brownian walks inside patches of targets with high density increases the search efficiency as compared to Lévy walks that does not react to the environment [12] [13].

Recent experimental data shows that the movement pattern of various marine predators, like blue basking shark (*Cetorhinus maximus*), bigeye tuna (*Thunnus obesus*) [14], and terrestrial animal like goat (*Capra hircus*) [8] shows Lévy walks pattern in areas with low abundance of preys or foods and Brownian walks in areas with high abundance. It is also argued in [7], that natural selection should favor flexible behaviors in animals, combining different searching strategies with different searching statistics under different conditions. Experimental data is also shown therein to prove that *Oxyrrhis marina* movement pattern follows Lévy and Brownian walks depends on the density of their prey (*Rhodomonas* sp.).

The underlying mechanism on how Lévy walks pattern is shown by animals is considered as an interesting topic. In relation with this, recently, it has been shown that animal foraging can also be subject to noise in the form of presumably internally-generated variability in an animal's choice of movements [4] [10] [15]. A foraging behavior mediated by internal noise has been reported in fruit fly Drosophila, causing them to perform Lévy walks pattern without any sensory input [4] [10], and zooplankton Daphnia, affecting the turning angle distribution to maximize the foraging success [15].

In order to realize Lévy walks pattern, one can surely use generated random numbers by sampling an approximation of Lévy stable distribution [16]. Simply transforming a uniformly distributed random variables to generate power law ones is also a commonly used method [12] [13]. Nevertheless, Gaussian noise is ubiquitous in nature due to the Central Limit Theorem, and it is reasonable to assume that efficient, and adaptive, foraging behavior is mediated by a natural Gaussian noise.

Furthermore, in [17], it is argued that animal movement pattern can sometimes be better characterized by different modes, as over long time-scales, simple models often fail to describe the patterns because of the likelihood that the animals change movement behavior. Similar idea is also described in [18]. In the work, a random walks model called the Lévy Modulated Correlated Random Walks (LMCRW) is proposed by incorporating a time discrete reorientation behavior with Lévy statistics, into the background continuous scanning process modeled as correlated random walks. In “reorientation” mode the turning angle is uncorrelated and breaks the directional persistence introduced by the “scanning” model, in which the turning angle distribution controls the persistence or correlation length of the random walks.

It has also been shown that such switching of modes in animal movement pattern can be adaptive to the environment. In relation with target density, in many natural environments the preys are actually clustered in patches: areas where the local resource density is higher than the mean overall resource density [19] [20]. As a result, various kinds of animals are observed to perform an adaptive switching between intensive search mode triggered by the detection of a prey within patches with high resource density, and an extensive search between these areas. This movement pattern is known as area restricted search (ARS), and reported to be performed by various animals, including some of those which adaptively choose Lévy or Brownian walks pattern based on target density: basking shark (*Cetorhinus maximus*) [21] and zooplankton (*Oxyrrhis marina*) [7].

Here, our focus of interest is the role of internal noise in realizing adaptive and efficient animal movement pattern. This paper presents a simple computational model based on natural Gaussian noise that can realize a mode switching between Lévy and Brownian walks movement pattern based on target density. Our approach is to borrow a model that attempts to explain Lévy walks pattern shown by one of the simplest creature, bacteria *Escherichia coli* [22]. We extend the model based on biological fluctuation framework, a recent perspective on how noise may be utilized in living beings [23] [24], without changing the essence of the stochastic property in *Escherichia coli* physiological mechanism.

To be more exact, the hypotheses that we would like to test in this paper are as follow: (1) Whether a behavior of choosing Lévy / Brownian walks movement pattern, each argued as the favorable strategy in a low / high target density, can be realized based on the model. (2) Whether in a commonly faced patchy environment where the target density alternates, an adaptive behavior will emerge based on the same model, and whether it can be beneficial.

To confirm the hypotheses, we built a simulation of a simple, generic animal whose movement follows the proposed computational model and analyze the behavior. We compare the realized behavior with the reported experimental data of zooplankton *Oxyrrhis marina*, the simplest animal that is shown to adaptively choose Lévy or Brownian walks under different target density, and confirm the similarity. We also perform simulation in a patchy environment using the same model and observe the realized behavior and efficiency.

In the next sections, we will first explain a more detailed knowledge related with the research. After that, the Method section will explain the proposed model. The Result section will show the simulation result that supports the hypotheses. In the Discussion section, the results are further discussed and compared with other well studied researches like the area restricted search, before the Conclusion is made.

### Lévy Walks and Brownian Walks

The term Lévy flights is used to describe a specialized random walks in which the flight lengths, the length between two consecutive change of direction, are drawn from a probability distribution with an inverse power-law tail [1] [2]:
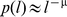
(1)with 1<*μ*<3, and *l* is the flight length. It means that rare but extremely long flight lengths can happen in the random walks pattern. Without tail truncation, sums of those flight lengths converge to a Lévy stable distribution. For *μ*≥3, there is no heavy tail in the distribution and the sums of the flight lengths converge to a Gaussian distribution due to the Central Limit Theorem, thus we recover Brownian walks. The case of *μ*≤1 does not correspond to distributions that can be normalized.

To be exact, a technically correct term is actually Lévy walks: a Lévy flight with time cost that depends on the flight lengths. A Lévy walk leads to anomalous diffusion, meaning that the mean squared displacement from the starting point increases faster than linearly with time *t*, while Brownian walks is a normal diffusion where the increase is linear.

As will be explained in the next section, the computational model is built based on *Escherichia coli* mechanism, which purely utilizes a natural Gaussian noise, with long correlation time as the key mechanism [22]. Thus, the model can be said as a family of fractional Brownian motion, a generalized form of Brownian motion with correlation time [25], and is indeed a model of correlated random walks. However, here we define the term based on the movement pattern, not the underlying process. We simply use the term “Lévy walks pattern” to describe a movement pattern with heavy tailed power law distribution, and “Brownian walks pattern” for movement pattern without such heavy tail.

It must also be noticed that the pattern are not generated by a Lévy process. Therefore, like common assumption about the Lévy walks pattern found in animals, the flight length distribution has a large, but finite variance [1]. It means after an extreme long period, the sum of causing the random search pattern to become a Brownian walks.

### Model of Escherichia Coli Motion

**Figure 1:**
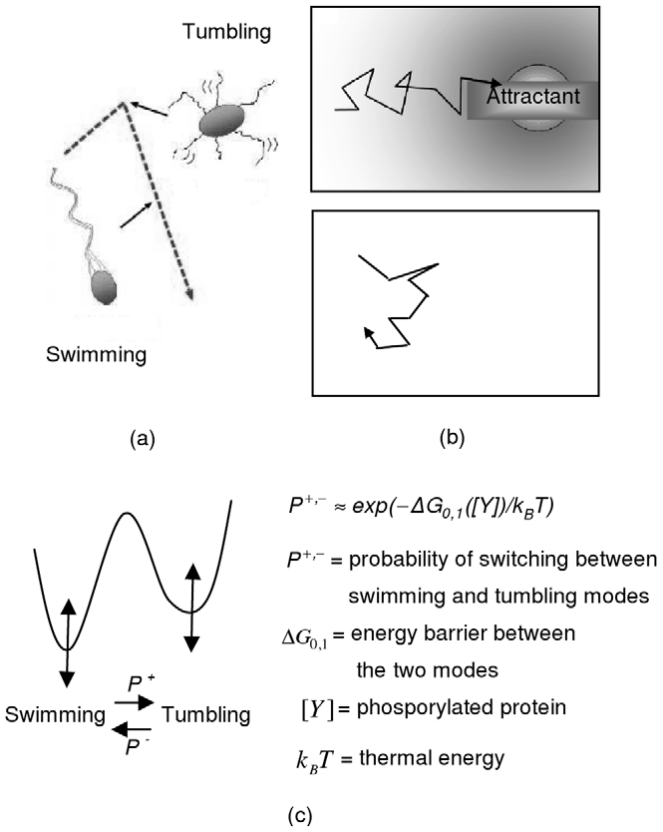
Model of *Escherichia coli* motion. (a) The two basic modes (b) A biased random walks with attractant, and random walks without any (c) The energy barrier model of switching probability between the two modes without attractant.

In bacteria, such as *Escherichia coli*, the motion can be characterized as a sequence of smooth - swimming runs, punctuated by intermittent tumbling motions that effectively randomize the direction of the next run [26] (Figure 1.a). The switching probability between the two modes is dictated by measurement of attractant chemical gradient in the environment, obtained from comparison of current and past concentration. When the bacterium perceives conditions to be worsening, the tendency to tumble is enhanced and vice versa. As a result, when the bacterium runs up a gradient of attractant, it will do chemotaxis, biased random walks toward the source. However, in the absence of this attractant, the bacterium will simply do random walks (Figure 1.b).

*Escherichia coli* motion is well studied in biology [22] [26]–[29] and engineering [30] [31]. However, only recently, unlike the conventional expectation that the swimming mode duration of *Escherichia coli* in absence of gradient follows Poisson-like distribution, a power law distribution is found [27]. A possible cause has been explained in [22]. It can be modeled that the switching probability between swimming and tumbling mode is an exponential function of an energy barrier [28], whose level keeps fluctuating due to Gaussian fluctuation of a phosphorylated protein concentration, Che Y-P inside the bacteria (Figure 1.c). By approximating the energy barriers by linear expansion as an average barrier value plus the protein fluctuation, it is shown that power law duration of the swimming mode, a Lévy walks pattern, can occur if the concentration of the protein fluctuates with a long correlation time. Equations (2) to (4) describe the explained dynamics in a more thorough way [21]. The dynamical two state models is shown by equation (2), while the linear expansion of the energy barriers is shown by equation (3) where α_0,1_ is two dimensionless constants characterizing the steepness of the response curves of the motor. Furthermore, equation (4) shows the simplest model of the dynamics of the CheY-P concentration where the first term shows the slow adaptation toward the preferred concentration [*Y*]_0_ with a correlation time *τ*, and *η*(*t*) is a Gaussian white noise, representing the fast stochastic driving force.
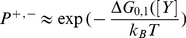
(2)

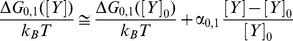
(3)

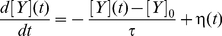
(4)


### Biological Fluctuation

Recent researches show that certain noise utilizing mechanism called “biological fluctuation”, or “Yuragi” in Japanese language, plays important role in various stages from molecules to brains in life sciences [23]. The mechanism is also found in bacteria adaptation to environmental changes by altering their gene expression when they are lack of certain nutrient. Based on this behavior, in [24] a simple model was built to explain the biological fluctuation. Here, the gene expression is modelled to be controlled by a dynamical system with some attractors. The model is also called “the attractor selection model” and represented by Langevin equation as:
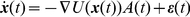
(5)where *x*(*t*) and −∇*U*(***x***(*t*)) are the state and the dynamics of the model at time *t*, with potential *U*(***x***(*t*)) can be designed to have some attractors *ε*(*t*) is the noise term. *A*(*t*) is a variable called “activity” which indicates the fitness of the state to the environment. From the equation, *U*(***x***(*t*))*A*(*t*) becomes dominant when the activity is large, and the state transition approaches deterministic. When the activity is small, *ε*(*t*) becomes dominant, and the state transition becomes more stochastic. The activity is therefore designed to be large when the state is suited to the environment and vice versa.

It should also be noticed that the dimension of ***x***(*t*) and what it represents depend on the phenomena tried to be modeled by equation (5), as shown by Yanagida et al [23]. For example, in [24], ***x***(*t*) consists of two variables that represent the concentrations of the mRNAs or their protein products, i.e. the gene expression. Furthermore, *A*(*t*) in their work represents “cellular activity”, a complex function of the concentrations of ATP and other chemicals, which is increased when cells approach the attractor, expressing the genes that allows survival and optimal growth in a given environment.

## Methods

In this section, we will explain the computational model based on the biological fluctuation framework, and the simulation setting to confirm the realized behavior.

### The Computational Model

In order to implement the “Yuragi” equation in (5) for realizing a Lévy and Brownian walks pattern based on target density, the first step is to properly choose the state of the attractor selection model. Borrowing the model from *Escherichia coli*, in which the probability of switching between the swimming and tumbling modes is an exponential function of energy barrier whose level keeps fluctuating changing due to fluctuation of certain chemical protein [22], we choose the state of attractor selection model *x*(*t*) in (5) as a representation of this protein fluctuation.

**Figure 2:**
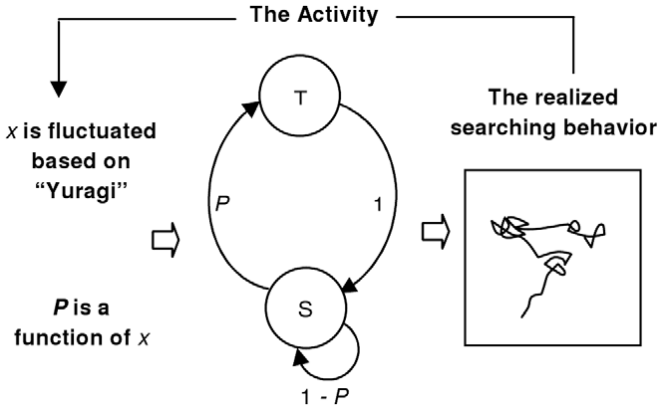
Model of Lévy and Brownian Walks Under Different Target Densities Based on Biological Fluctuation. Variable “*x*” represents fluctuation of internal variable like the pohosporylated protein in *Escherichia coli*. The center figure is a probabilistic state machine between *S* = swimming and *T* = tumbling mode that represents motion of moving forward and change direction randomly, where *P* is a function of *x*. The activity indicates whether the current searching behavior is suited to the current target density, affecting fluctuation of *x*.

To make the model clearer, one can draw a probabilistic state machine as shown in Figure 2 (center). Here, “*P*” equals to “*P^+^*” in Figure 1.c, which is the tumbling probability (i.e. switching from swimming to tumbling mode). The swimming mode is defined as moving forward with a certain distance, while tumbling means changing direction randomly. In this paper, the swimming probability (i,e, switching from tumbling to swimming mode), or “*P^−^*” in Figure 1.c, is simply considered as 1. It is the simplest case which assumes the animal stops turning at the next time step. This suits our purpose, as in this paper we will only confirm the relationship between the search efficiency and the flight length distribution. While it is possible to investigate the effect of the turning angle characteristics to the efficiency like conducted in some researches (e.g. [32] [33]), such aim is outside of the scope of the current paper.

While other function may actually be used, borrowed from *Escherichia coli* model, here we relate *P*(*t*) and *x*(*t*) by an exponential function :
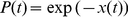
(6)To further explain the relationship with *Escherichia coli* mechanism, by approximating the barrier fluctuation by linear expansion, as shown in (3), one can rewrite (2) into (7a) and (7b) as follows:
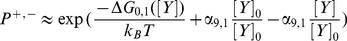
(7a)

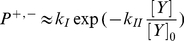
(7b)where:
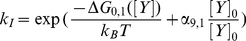
(8a)

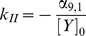
(8b)It can be seen that in our model, *x*(*t*) clearly represents [*Y*](*t*), the phosphorylated protein concentration, Che Y-P, inside the bacteria, with *k_I_* and *k_II_* chosen as 1 for simplicity. While the parameter values may change the property of the movement pattern (i.e. the exponent value), they will not change the essence of the stochastic process. Furthermore, in *Escherichia coli*, due to an assumed reasonable value of these parameters, unlike the swimming duration, the tumbling duration supposes to follow an exponential distribution [22], meaning that “*P^−^*” in Figure 1.c approximates certain constant value. In our model, it can be seen that this constant value is assumed as 1, for the reason explained in the previous paragraph.

Following the *Escherichia coli* behavior, Lévy walks pattern supposes to happen when *x*(*t*) fluctuates with a long term correlation [22]. However, shorter correlation time supposes to realize a less correlated random walks, with the sum of those flight lengths converge to Gaussian distribution, i.e. a Brownian walks.

In order to investigate whether a Lévy and Brownian walks pattern can emerge based on target density, we design the dynamic of *x*(*t*) based on (5), following a simple unimodal potential function *U*(*x*(*t*)) shown below:
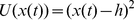
(9)causing the dynamics of *x(t)* shown in (5):
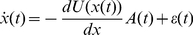
(10a)

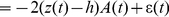
(10b)


The first term in (10) represents slow adaptation toward a preferred value of *h*, which corresponds to the attractor. The noise term, *ε*(*t*) is zero mean Gaussian white noise, represents the stochastic driving force similar to the Gaussian internal protein fluctuation in bacteria [22]. The activity *A*(*t*) changes the shape of the potential *U*(*x*(*t*)) and therefore correlation time of state *x*(*t*), as the key mechanism of the model.

From (10a), it can be seen that small value of *A*(*t*) will cause *U*(*x*(*t*))*A*(*t*), potential *U*(*x*(*t*)) multiplied by *A*(*t*), to be flat and variable *x*(*t*) supposes to fluctuate with long correlation time. Therefore, Lévy walks pattern in an animal trajectory with certain power law exponent in (1): 1<*μ*<3, supposes to be realized. When the activity *A*(*t*) has a large value, the shape of *U*(*x*(*t*))*A*(*t*) supposes to be sharp to let variable *x*(*t*) fluctuate with short correlation time. Therefore, Brownian walks pattern supposes to be realized, with the exponent *μ* equal or larger than 3.

To let Lévy and Brownian walks pattern emerges based on target density, the activity is defined as a function of sensory input. When no targets are found, the activity should be low such that the shape of *U*(*x*(*t*))*A*(*t*) is adequately flat and Lévy walks is performed. However, once some targets are found, the activity should be high, and the shape of the *U*(*x*(*t*))*A*(*t*) should become sharp, supposedly reduces the correlation time, causing the Lévy walks to switch to Brownian walks pattern. Here, the activity function can be summarized in equation (11) to (13).
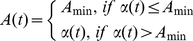
(11)

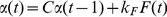
(12)

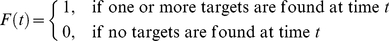
(13)


Anytime one or more targets are found at time *t*, *F*(*t*) will be triggered to 1. It can therefore be said that *F*(*t*) is a step function whose input is the finding of targets. The definition of finding the target will be explained in the next section. Furthermore, *α*(*t*) is the running average of the number of targets found as shown in (12), with 0<*C*<1, while *k_F_* is a constant with a large value in comparison to *A_min._*. By employing such function, the correlation time will be reduced when a target is found and gradually increases to the original value, if no more targets are found. The overall principle is shown in Figure 2.

In *Escherichia coli*, as the movement is dictated by measurement of attractant chemical gradient in the environment, one can easily see that in order to model such movement, the activity function should be a function of the gradient.

### The Simulation Setting

In order to understand how the simulated animal will behave, at first, we observe the generated random walks without sensory input, and give a range of constant values for the activity. The position of the attractor is chosen as *h* = 0.7, corresponds to *P*(*t*)≈0.5 according to (6) when *x*(*t*) is entrained into the attractor. In order to let *P*(*t*) adequately fluctuates between 0 and 1 as a function of *x*(*t*), the value of *x*(*t*) is limited between 0 and 5, corresponds to those values of *P*(*t*). The size of noise *ε*(*t*) in (10) is 0.5, defined by the standard deviation. The simulated time is 10000 [s]. Equation (10) is discretized with time sampling 0.1 [s] and the state *x*(*t*) transits to the next state *x*(*t*+1) at each time step, changing the tumbling probability from *P*(*t*) to *P*(*t*+1). For the swimming mode, the length of moving forward is defined as 1 [unit]. As here we focus on the flight length distribution, for simplicity, the random turning angle in the tumbling mode is set to be uniformly distributed from 0 to 360 [deg]. To analyze the behavior, as the first step, we observe the relationship between the value of the activity and the generated random walks. We want to confirm whether at certain values of the activity, which can be corresponded to the target density by the activity function, the animal will show Lévy and Brownian walks pattern. From the chosen range of the activity value, we observe the effect to the shape of *U*(*x*(*t*))*A*(*t*), to the correlation time of state *x*(*t*), and the realized random search pattern performing by the animal.

**Figure 3:**
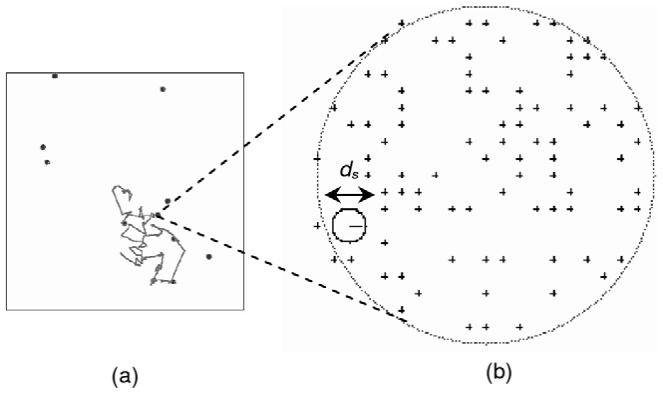
The simulation screenshot for the patchy environment. (a) From a usual scale, and (b) The zoomed-in condition when the animal enters a patch of targets.

As the second step, to further confirm the behavior under changing target densities, we want to investigate whether an adaptive switching between Lévy and Brownian walks pattern will emerge in a patchy environment and analyze its benefit. We will also discuss the behavior from the perspective of area restricted search behavior. In order to do that, we create the patchy environment setting, and Figure 3 shows the screenshot of the created simulation environment from two different scales. Figure 3 (a) shows the screenshot of the whole area. The size of the area is 1000×1000 [units]. The targets inside the patch are not shown for clarity. We deploy the simple, generic animal at the center of the screen at the beginning of the simulation. Here, we use periodic boundary condition, a common approach used in observing random search performance [13], which means once the animal passes the simulation boundary, it will reappear from the other end. This will present an experiment result that is unaffected by different boundaries of the area, and, with long enough simulation time, where the animal starts the search.

Figure 3 (b) shows the zoomed in condition when the animal approaches some targets, shown by the crosses. One or more targets are considered to be found, and disappear at the next time step, if their position is inside the animal sensing diameter, *d_s_*, representing a limited sensing capability of the animal. The Lévy walks pattern has been shown to be better when the targets are sparse, that is the target site has a low target density. In other word, the average distances among the targets are much larger than this diameter. However, Brownian walks pattern is supposed to be favorable inside patches with high target density. If the sensing diameter is infinite, then the animal does not have to do any search as it will automatically find all of the targets regardless where the animal is. For further study, one can refer to [1] [2] [11]. Here, our focus is to investigate whether based on the model, the animal can have properly choose the more suitable random search under different target density.

In creating the patchy target setting, we make sure that the patches are sparsely placed while each patch has higher target density. One can easily see that if the patches are not sparsely placed then it cannot be called patchy environment as the overall target density will become high, in which Brownian walks is shown to be favorable [11]. On the other hand, if each patch only contains a few targets then it cannot be called patchy environment either, as the overall target density will become low in which Lévy walks is the better strategy [1] [2]. Therefore, we deploy 10 circular shape patches with a small radius of 10 [units] in the 1000×1000 [units] search area. To make sure that each patch is dense, 100 targets are deployed inside each of them. The animal sensing diameter *d_s_* is 2 [units]. The simulation screenshot showing the patches, the targets inside them, and *d_s_* can be seen from Figure 3. While investigating the behavior in a more varying target distribution is a part of our future works, here we simply make sure that the target density is changing and set the targets inside the patches and the center of each patch in the whole area to be uniformly distributed.

To measure the performance of the search, we observe the search efficiency, defined as the number of targets found divided by total distance travelled. The criteria is related with the energy efficiency, as moving forward generally takes more energy than changing direction randomly, therefore also used widely in animal random search literatures [11]–[13].

To realize the an adaptive switching between Lévy and Brownian walks in a patchy environment, we choose a certain minimum value of the activity *A_min_*, and implement the activity rule explained in (11) to (13) to change the random search depends on target density. The performance of this adaptive search is then compared with Lévy and Brownian walks alone, and discussed afterward from the perspective of area restricted search behaviour.

For every experiment, we perform 20 trials, and use the commonly known t-test if it is necessary to confirm a statistical significance of a data comparison.

## Results

Here, we observe the generated random walks with different value of the activity, which can easily be corresponded to different target densities, as well as the realized adaptive behavior in a patchy environment.

### Generated Random Walks with Different Value of the Activity

We observe fluctuation of *x*(*t*), its correlation time and the resulting value of *μ* with a range of the activity *A*(*t*) values. To determine whether the realized searching behavior follows a Lévy or Brownian walks pattern, the most common approach is to plot the log-log histogram of the flight's frequency *N*(*l*) versus the lengths *l*. The frequency is normalized (i.e. divided by the histogram bin width and total frequency), while the bins are increased logarithmically. Because the minimum value of *l* is one, here we use bin breaks of 1,2,4, and so on. Power law statistic is indicated if a straight line fits the plotted data. The method is called “LBN” (logarithmic binning with normalization), recommended in [34], and actually the slope of the fitted line will be equal to minus of the power law exponent, *μ* in (1). However, the accuracy of the approach is criticized [3] [35], therefore we reconfirm the power law statistic over exponential by using a maximum likelihood method explained in [35], and calculates the value of *μ* using a more recent, accurate, method explained therein, shown in (9), where *L* is the whole data set of the flight lengths. The equation can be used because the minimum value of the flight length is 1. A Lévy walks pattern will be shown if 1<*μ*<3.
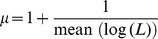
(9)


In order to observe the correlation time of state *x*(*t*), we plot the autocorrelation function, defined as *R*(*x*(*t*)), and calculate the correlation time, *t_RX_*. We use the common definition of correlation time, that is the time when the autocorrelation value of the state is already at a factor of *1/e* down from its maximum value at *t* = 0 [36].

**Figure 4:**
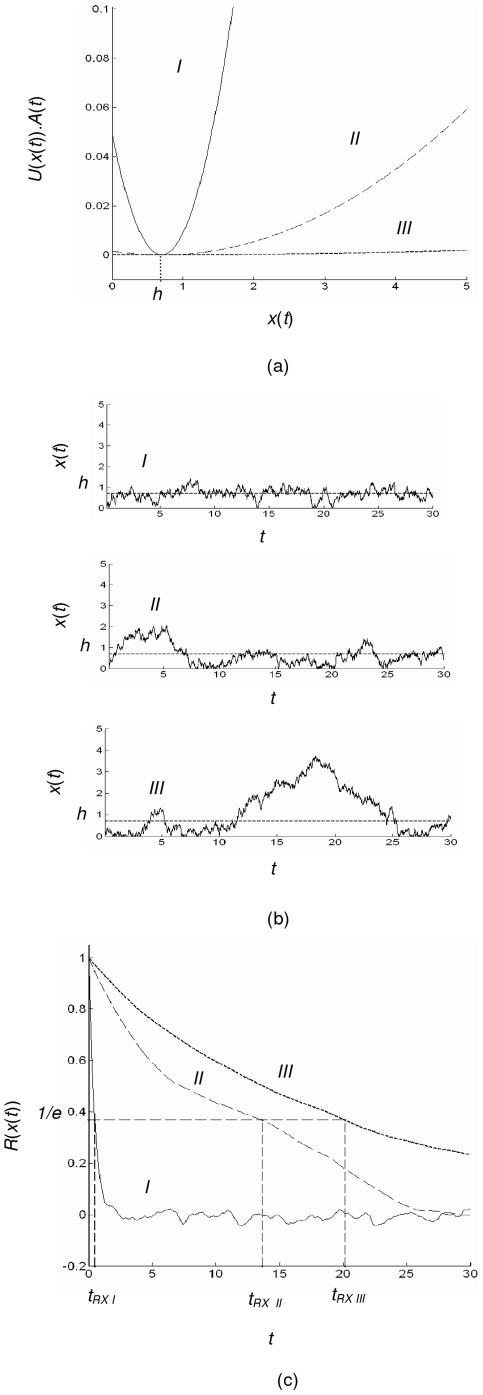
Dynamics of the model. (a) The shape of potential *U*(*z*(*t*)).*A*(*t*) (b) The resulting fluctuation of x*(t)* around the attractor at *h* = 0.7 (c) The autocorrelation function *R*(*x(t*)) with the correlation time *t_RX_* I, II, III corresponds to *A*(*t*) = 10^−1^, 10^−2.5^, 10^−4^ consecutively.

**Figure 5:**
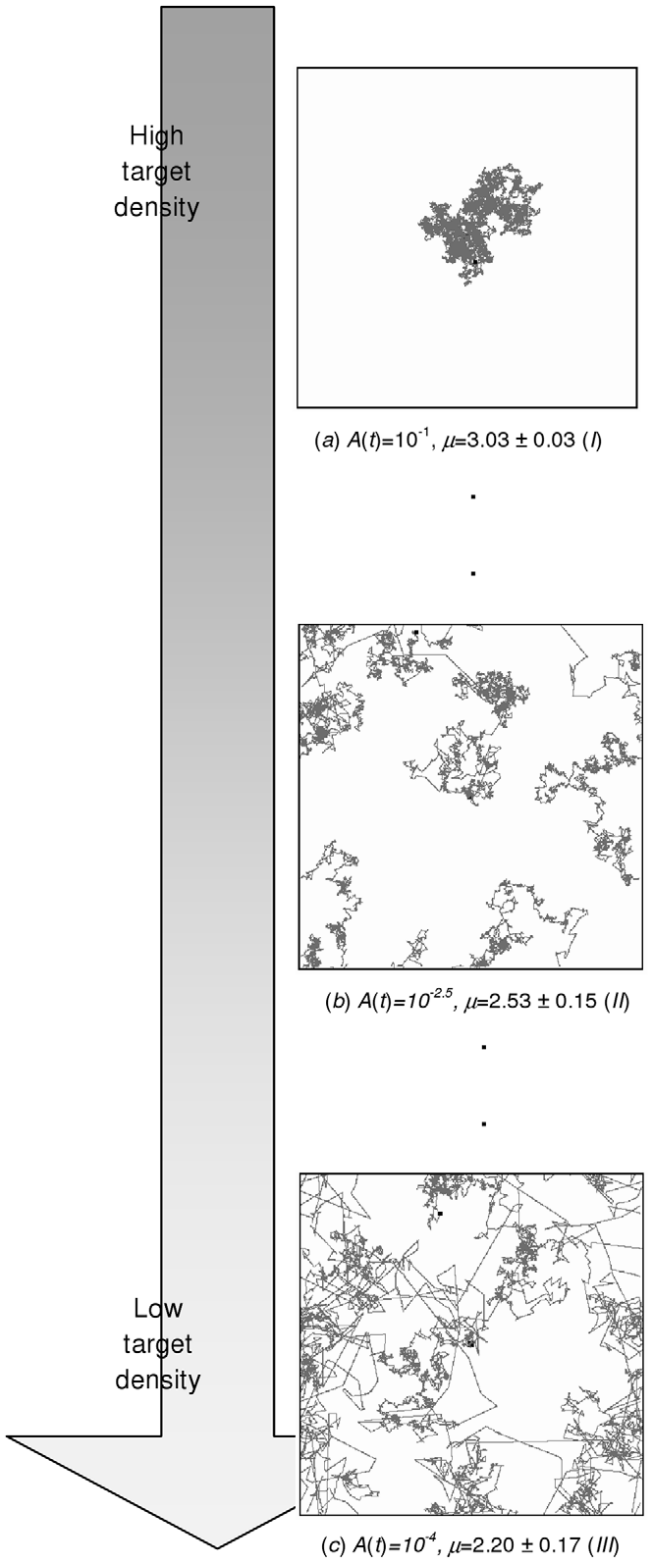
The corresponding trajectory examples. The figure also shows the values of the activity with the average calculated value of exponent *μ*, along with the corresponding target density if the activity function is designed such that the activity value is be proportional to the to the target density.

Figure 4 (a), (b) and (c) show the corresponding shape of potential *U*(*x*(*t*)) multiplied by the activity *A*(*t*), the resulting example of fluctuation of *x*(*t*) along with the autocorrelation graph with shown position of *t_RX_* in the first 25 [s], with the value of the activity that corresponds to the trajectory shown in Figure 5. It can be seen that with small value of the activity, the potential *U*(*x*(*t*))*A*(*t*) is flat and let *x*(*t*) fluctuate with large correlation time *t_RX_*. On the other hand, with large enough *A*(*t*), *x*(*t*) fluctuates around the position of attractor *h* = 0.7 with a small correlation time *t_RX_*.

Figure 5 shows the realized trajectories for certain values of the activity, with the resulting exponent *μ* that indicates whether the animal performs Lévy or Brownian walks. It can be seen that the value of the activity, which controls the effect of the noise, controls the random walks behavior of the animal. Therefore, it is easy to see that by properly choosing an activity function such that the activity value is proportional to the target density, in areas with a high target density (e.g. there are abundant numbers of patches) or a low density (e.g. there are only a few patches with a few targets inside them), the animal would do Brownian and Lévy walks consecutively, the argued better strategy for those conditions. This is caused by the value of the activity that most of the time will likely be high for the first condition, and never increase significantly for the second one.

**Figure 6:**
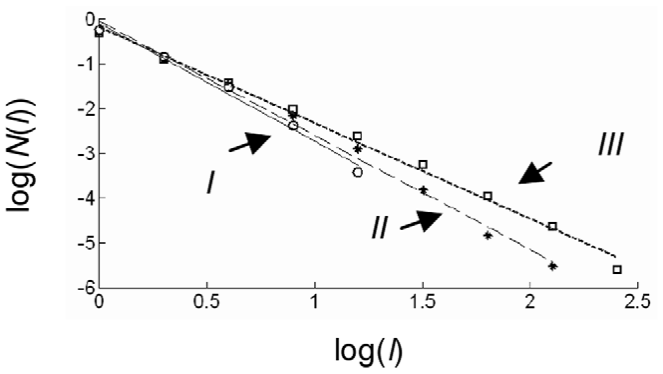
The corresponding Log-log histogram. Log-log histogram and the approximated fitted line of normalized flight lengths frequency *N(l)* versus the flight lengths *l* for each activity value shown in Figure 6.

Furthermore, Figure 6 shows examples of the log-log graph of flight lengths frequency *N*(*l*) versus the flight lengths *l* and an approximated straight line that indicates power law statistic.

**Figure 7:**
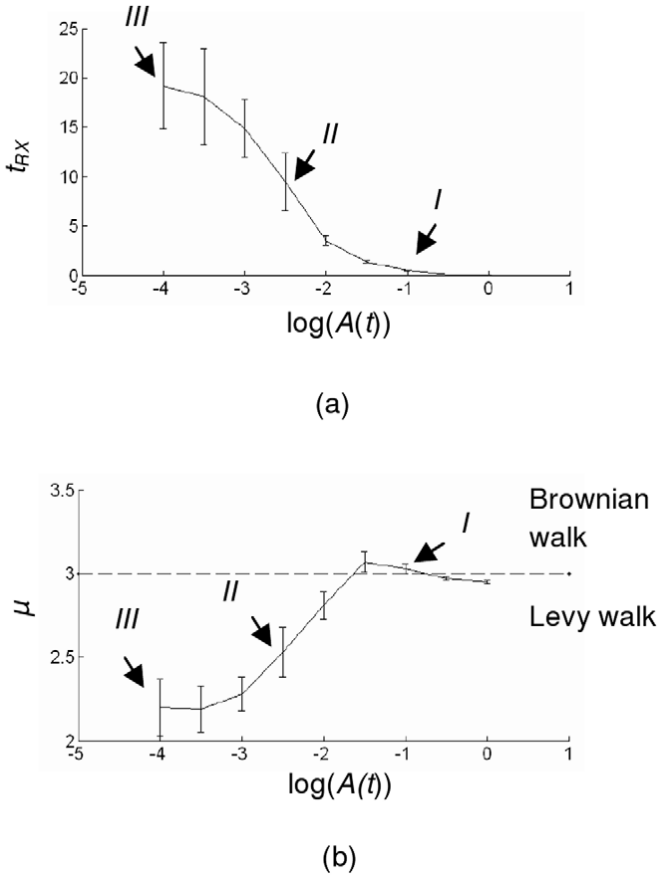
The relationship between activity, correlation time and power law exponent. (a) Correlation time *t_rz_* versus the activity *A*(*t*) (b) Exponent *μ* versus the activity *A*(*t*). For clarity, the activity is plotted in a log scale.

Figure 7 explains the behavior of the random search in a more thorough way. The figures show the relationship between log of the activity *A*(*t*) versus the correlation time *t_RX_* (a) and exponent *μ* (b). The vertical bars show the standard deviation. It can be seen clearly that when the activity has a small value, causing a long correlation time, the animal will do a Lévy walks pattern. When the activity gets larger, the correlation time is reduced and the random search will have a stronger tendency to become a Brownian walks. When the activity equals to 1, the value of *μ* already about equals to the condition if *x*(*t*) is simply kept constant at *h*.

To compare the result with the reported data, Table 1 shows the power law exponent data of the representative 2D trajectory pattern of *Oxyrrhis marina* with different densities of *Rhodomonas* sp. preys reported in [7]. It can be seen that the behavior can be imitated by defining the activity function such that the activity value is proportional to the targets or preys density.

**Table 1:** The Lévy and Brownian walks pattern found in *Oxyrrhis Marina* under different prey (*Rhodomonas* sp.) densities reported in [7].

Power law	Density of Preys
Exponent (*μ*)	
**>3.0**	High (1×10^4^ to 1×10^5^ cells per ml)
**(exponential)**	
**2.1/2.2** *	Medium (1–2×10^3^ cells per ml)
**2.1/2.2** *	Low (1×10^1^ to 5×10^2^ cells per ml)

* (‘/’ separates the different results between the first and second experiment performed in [7]).

### Adaptive Search in a Patchy Environment

To further investigate the behavior of the animal based on the model under different target densities, it is interesting to observe the emergent behavior a natural patchy environment where the target density alternates, and to see whether it can be beneficial. In order to investigate this, we implement the activity rule in (11) to (13) with *A_min_* = 10^−4^ whose properties indicated by the number *III* in Figures 4, 5, 6, and 7 such that the animal will do Lévy walks when no targets are found, that is when the target density is likely to be low. The constant *k_F_* is set to 10^−1^ such that when some targets are found and *F*(*t*) in (12) equals to 1, the animal will immediately switch to a Brownian walks whose properties indicated by the number *I* in Figures 4, 5, 6, and 7. When the local target density is high, it is likely that the animal will keep finding another target such that the value of the activity will be kept high and the animal will keep dong Brownian walks. When no more targets are found, the animal will gradually switch back to Lévy walks with the activity *A*(*t*) equals to 10^−4^. The value of constant *C* in (12) is 0.9.

**Figure 8:**
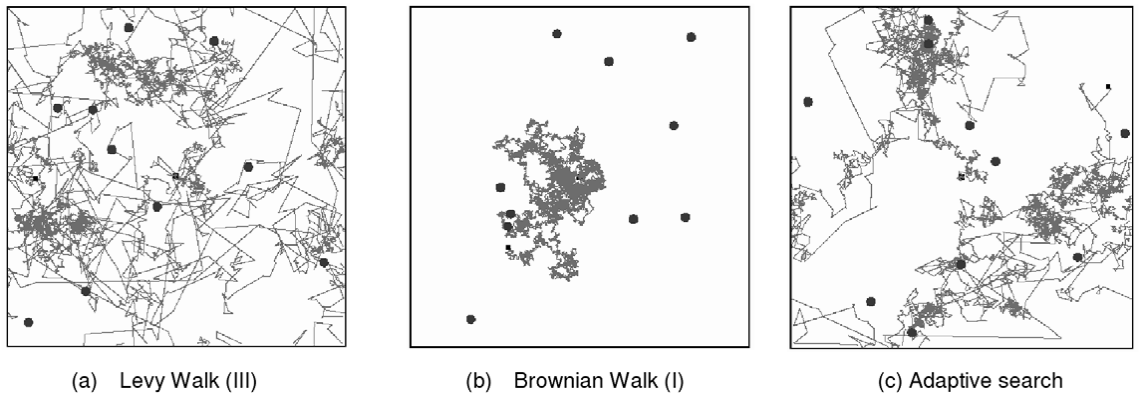
Examples of the realized trajectories in a patchy environment. (a) Lévy Walks (I) (b) Brownian Walks (III) (c) Adaptive search.

The trajectory comparison between Lévy, Brownian walks and the adaptive search is shown in Figure 8. It can be seen that due to the occasional long flight lengths, as variable *x*(*t*) sometimes fluctuates near a high value as shown in Figure 4.b (bottom), the Lévy walks pattern finds more patches than the Brownian walks. This causes higher search efficiency even that the total traveled distance is also a little bit higher. However, it is interesting to notice that while Brownian walks finds less number of patches, the ratio between targets found and visited patches are actually higher, as the search is more intensive when a patch is found. This indicates that switching between the two random search behaviors might be beneficial. It is confirmed in Table 2, which shows the mean and standard deviation of the efficiency, along with other criteria. It can be seen that performing the adaptive behavior is better than either Lévy or Brownian walks alone. The statistical significance of this result has been confirmed by using t-test.

**Table 2:** The properties of Lévy Walks, Brownian walks and the adaptive search in a patchy environment.

Properties	Lévy Walks	Brownian	Adaptive
	(III)	Walks (I)	Search
			(*C* = 0.9)
**Search**	1.54±0.73	0.86±0.82	2.78±1.10
**efficiency**			
**Targets**	(1.32±0.65)	(0.57±0.55)	(2.23±0.83)
**found**	×10^2^	×10^2^	×10^2^
**Travelled**	(8.48±0.26)	(6.67±0.02)	(8.08±0.27)
**distance**	×10^4^	×10^4^	×10^4^
**Visited**			
**patches**	5.25±1.80	1.45±1.15	4.75±1.16
**(out of 10)**			
**Average**			
**activity**			
**value**	10^−4^	10^−1^	(1.14±0. 27)
**inside**			×10^−1^
**patches**			
**Average**			
**activity**			
**value**	10^−4^	10^−1^	(5.03±1.35)
**outside**			×10^−4^
**patches**			

From the trajectories in Figure 8, it can be seen that, unlike the Lévy walks which does not react when a target is found, in the adaptive search, the intensive searches are concentrated near the patches. This behavior can be explained by comparing the average activity value of Lévy and the adaptive search shown in Table 2. It can be seen that for the adaptive search, the average activity inside the patch, in a log scale, corresponds to the exponent *μ* for a Brownian walks pattern shown in Figure 7, confirming the switching behavior. However, outside these patches, the value of the activity is also slightly higher as it takes sometimes for the animal to gradually switch back to Lévy walks after it does not find any targets for certain period. As a result, the exponent *μ* outside the patch will have a slightly higher value, according to Figure 7 (b).

Parameter *C* in (7) decides how strong the tendency to switch back to Lévy walks (see Table 3). Too small value of *C* is meaningless, as it will not be able to increase the value of the activity large enough to make the animal adequately switch to Brownian walks inside the patches. On the other hand, too large value of *C*, may cause a longer time to switch back to the Lévy walks pattern. For the used patchy environment, *C* = 0.9 is shown to be the best value.

**Table 3:** The properties of the adaptive search with different value of the parameter *C* in (7).

Properties	*C* = 0.99	*C* = 0.9	*C* = 0.5
**Search**	2.33±1.07	2.78±1.10	1.69±0.63
**efficiency**			
**Targets**	(1.81±0.82)	(2.23±0.83)	(1.41±0.51)
**found**	×10^2^	×10^2^	×10^2^
**Travelled**	(7.81±0.15)	(8.08±0.27)	(8.42±0.27)
**distance**	×10^4^	×10^4^	×10^4^
**Visited**			
**patches**	4.05±1.54	4.75±1.16	4.90±1.33
**(out of 10)**			
**Average**			
**activity**			
**value**	(5.52±1.30)	(1.14±0. 27)	(0.50±0.16)
**inside**	×10^−1^	×10^−1^	×10^−1^
**patches**			
**Average**			
**activity**	(78.73		
**value**	±32.31)	(5.03±1.35)	(1.17±0.08)
**outside**	×10^−4^	×10^−4^	×10^−4^
**patches**			

The results explained in this Result section confirm the hypotheses that the model can realize a behavior of choosing Lévy or Brownian walks pattern depends on target density. It has also been confirmed how an adaptive behavior of switching between the two random walks will emerge in a commonly faced patchy environment, and how it can be beneficial.

## Discussion

In this paper, we have presented a simple, Gaussian-noise utilizing, computational model that can imitate animals behavior of performing Lévy and Brownian walks depends on the target density. The model proposes two important concepts to realize a noise utilizing searching behavior: the activity rule that regulates the fluctuation of certain internal variable(s); and the attractor(s) where the variable is entrained to when the value of the activity is high.

The proposed model has advantages of universality and simplicity, as it can be described based on two most basic motions: moving forward (swimming) and changing direction (tumbling), and built based on a natural Gaussian fluctuation. By principally controlling the correlation time of tumbling probability fluctuation through the activity rule, and relates the rule to target density, we have shown that based on the model, Lévy walks or Brownian walks pattern will be realized depends on the target density.

An interesting question of course is whether the used activity rule that enables the animal to perform such adaptive behavior has similarity with the physiological mechanism of *Escherichia coli*. By comparing equation (4) and (10), it can be seen that the simplest model of the dynamics of the CheY-P concentration can be described by attractor selection model with the preferred concentration of CheY-P, [*Y*]_0_, as a single attractor. It is also known that CheY, a response regulator protein in bacterial chemotaxis, in its active, phosphorylated form, exhibits enhanced binding to a switch component at the *Escherichia coli* flagellar motor which induces a change from counterclockwise (CCW) to clockwise (CW) flagellar rotation and determines the swimming behaviour [37]. Through this mechanism, in the presence of chemoattractant, the tumbling probability will be suppressed, enabling the bacteria to climb up the attractant gradient [38].

While the relationship between CheY-P concentration and the swimming behaviour of bacteria inside attractant can be explained in a more detail manner [38], equation (10) adequately describes this behaviour. It can be seen that when the activity has a high value, *x*(*t*) will be entrained to the attractor, suppressing the tumbling probability into certain value. The difference here is that the suppressing of tumbling probability is not caused by a gradient of chemoattractant, but by an increasing number of targets found. However, they are similar because the concentration of the chemicals is identical to the number of molecules in certain area. In order to model *Escherichia coli* behaviour of climbing up certain attractant gradient, one can easily change the equations that relate the activity with sensory input in equation (11) to (13), to be a function of the gradient instead of the targets found.

It is also interesting to compare the model with other model that attempts to describe a mode switching behaviour in animal. For example, in LMCRW, in the “scanning” mode, after each step, the turning angle is correlated with the previous one and controls the persistence of the random walks [17]. In this model, such behaviour can be realized if the definition of the swimming mode is changed such that the animal also changes the direction according to certain correlated turning angle distribution after one step of moving forward. *Escherichia coli* itself swims in a relatively smooth, straight line [29]. Therefore, the turning angle at the end of every time step in the “scanning” mode can be ignored if the movement is to be modeled by the LMCRW, similar to the condition considered in this model. The tumbling mode explained in the model is similar to the “reorientation” mode in LMCRW in which the animal changes direction randomly with uncorrelated turning angle.

We have also observed the emergent behaviour based on the same model in a commonly faced patchy environment, meaning that the target density alternates. As expected, the simulated animal will adaptively switch between Lévy and Brownian walks. It is also confirmed that such behaviour is able to increase the searching efficiency, supporting the previously reported results with similar theoretical model that does not based on Gaussian fluctuation [12] [13]. We have also explained how the parameter in the activity rule will affect the switching behaviour and how it relates with the increase of the searching efficiency. This theoretical result is interesting, as it suggests that animals that are able to properly choose Lévy and Brownian walks depends on target density will behave efficiently in a commonly faced patchy environment.

As have been explained in the Introduction section, another perspective to explain behaviour pattern of animals in a patchy environment is area restricted search (ARS) [19] [20]. It is therefore an interesting question how to explain the adaptive searching behaviour from ARS perspective. Following ARS explanation as encountered random walks, that is animal remains in a slow-diffusion state for certain period following an encounter with target, before transiting to a fast-diffusion state [20], the model clearly realizes ARS as its emergence behavior under a patchy environment. This is due to the change between fast, non linear, diffusion of Lévy walks as the extensive search and slow, linear, diffusion of Brownian walks as the intensive search.

A change of modes is actually not necessary to create a movement pattern that can be categorized into ARS. A Lévy walks pattern with certain exponent value (e.g. *μ* = 2) is enough to produce a pattern that is similar to ARS. In this model, it can be seen that such pattern is created because the turning probability keeps fluctuating between high and low value, which makes the animal sometimes alternate between extensive and intensive search even without encountering targets. However, once the animal enters a patch of targets, the turning probability will be entrained to certain value, triggering an intensive search inside the patches.

Indeed, aside from Lévy, Brownian or combination thereof, there are also other ways to stochastically model animal movement, such as the Lagrangian approach which is also based on stochastic differential equation (for a nice review, one can refer to [39]). However, to the author knowledge, most of the approaches focus on directly modeling how the position of the animal changes in real physical space, rather than trying to build a model based on certain assumption of the internal mechanism.

This paper focuses on the role of noise in such internal mechanism to realize an adaptive and efficient behavior. In relation with this, stochastic resonance, which has been studied extensively in the context of sensory system [40], in its most general form can be said as a process whereby the addition of random function or ‘noise’ can optimize a physical or biological process. It is interesting to notice that the described model has similarity with the concept of stochastic resonance in a sense that an addition of certain amount of noise will increase the system performance. To be more exact, without the noise term in equation (10), the flight length distribution will simply becomes exponential, causing a Brownian walks, forever. As have been shown, this can decrease the search performance significantly.

In addition to the flight length distribution, it may also interesting to discuss how the parameters of the model should be tuned to better characterize movement of certain animals, in addition to the flight length distribution. For example, while it is confirmed that in areas with high density of their prey (*Rhodomonas* sp.), zooplankton *Oxyrrhis marina* follows a Brownian walks pattern, this pattern looks more like a long straight paths with crossover (the first figure in reference [7]), meaning that the probability of changing direction is likely to have a low value. Based on the model described in this paper, the above explained behaviour can be realized by assigning a higher value to the attractor, such that when the activity has a high value, the probability to tumbling will be entrained to a low value.

It is also an intriguing question whether the same animals that performs Lévy and Brownian walks pattern under different target density will also do a certain area restricted search behavior in a patchy environment, and whether their behavior can be explained based on the same, possibly noise utilizing, model. As it is common for animals to perform area restricted search, including basking shark (*Cetorhinus maximus*) [17] and zooplankton (*Oxyrrhis marina*) [7] that is shown to adaptively choose Lévy and Brownian walks under different target density, to further investigate this issue is an interesting future work.

It has been argued in [7] that the searching behavior of individuals is, at least in part, genetically encoded, and therefore we should expect natural section to favor flexible searching statistic in animals under different conditions. Such argumentation is also mentioned in [18], emphasizing more in the advantage of noise in optimizing biological process, and therefore the noise should have been internalized by natural selection, which is indeed supported by experimental data [4] [10] [18]. The biological fluctuation framework is aimed to model such internal noise utilizing mechanism. Here, in principle we have shown how a widely attracting animal searching behavior can be modeled based on it, in a simple way. It is an interesting direction to see how the framework may be expanded for further investigation on the role of noise in realizing an adaptive and efficient searching behavior in animals.

### Conclusion

This paper presents a simple, Gaussian noise utilizing, computational model that can imitate animal behavior of performing Lévy and Brownian walks pattern in a low and high target density. Based on the biological fluctuation framework, the model is proposed without changing the essence of the stochastic property of the Lévy walks model of one of the simplest creature, *Escherichia coli*. In a patchy environment where the target density alternates, we have also observed the emergent behavior of adaptively switching between Lévy and Brownian walks and confirm the benefit. We have also explained how the parameters of the model will affect the realized behavior, and discussed the comparison with existing well studied models as well as the research direction motivated by the work.
